# Effects and mechanisms of Zhizi Chuanxiong herb pair against atherosclerosis: an integration of network pharmacology, molecular docking, and experimental validation

**DOI:** 10.1186/s13020-023-00874-x

**Published:** 2024-01-11

**Authors:** Yan Zhang, Yifei Qi, Zijun Jia, Yiming Li, Liqi Wu, Qingbing Zhou, Fengqin Xu

**Affiliations:** 1 Institute of Geriatrics, Xiyuan Hospital, China Academy of Chinese Medical Sciences, Beijing, China; 2 Comprehensive Care of China-Japan Friendship Hospital, Beijing, China; 3 Department of Integrated Traditional and Western Medicine, Peking University, Beijing, China

**Keywords:** Atherosclerosis, Macrophage polarization, Zhizi Chuanxiong herb pair, TNF/NF-κB axis, Network pharmacology, Molecular docking

## Abstract

**Background:**

The Zhizi Chuanxiong herb pair (ZCHP) can delay pathological progression of atherosclerosis (AS); however, its pharmacological mechanism remains unclear because of its complex components. The purpose of current study is to systematically investigate the anti-AS mechanism of ZCHP.

**Methods:**

The databases of TCMSP, STITCH, SwissTargetPrediction, BATMAN-TCM, and ETCM were searched to predict the potential targets of ZCHP components. Disease targets associated with AS was retrieved from the GEO database. Gene Ontology and Kyoto Encyclopedia of Genes and Genomes (KEGG) signaling pathway analyses were executed using DAVID 6.8. Molecular docking method was employed to evaluate the core target binding to blood components, and animal experiments were performed to test action mechanism.

**Results:**

A ZCHP-components-targets-AS network was constructed by using Cytoscape, included 11 main components and 52 candidate targets. Crucial genes were shown in the protein–protein interaction network, including TNF, IL-1β, IGF1, MMP9, COL1A1, CCR5, HMOX1, PTGS1, SELE, and SYK. KEGG enrichment illustrated that the NF-κB, Fc epsilon RI, and TNF signaling pathways were important for AS treatment. These results were validated by molecular docking. In ApoE^−/−^ mice, ZCHP significantly reduced intima-media thickness, pulse wave velocity, plaque area, and serum lipid levels while increasing the difference between the end-diastolic and end-systolic diameters. Furthermore, ZCHP significantly decreased the mRNA and protein levels of TNF-α and IL-1β, suppressed NF-κB activation, and inhibited the M1 macrophage polarization marker CD86 in ApoE^−/−^ mice.

**Conclusion:**

This study combining network pharmacology, molecular biology, and animal experiments showed that ZCHP can alleviate AS by suppressing the TNF/NF-κB axis and M1 macrophage polarization.

**Graphical Abstract:**

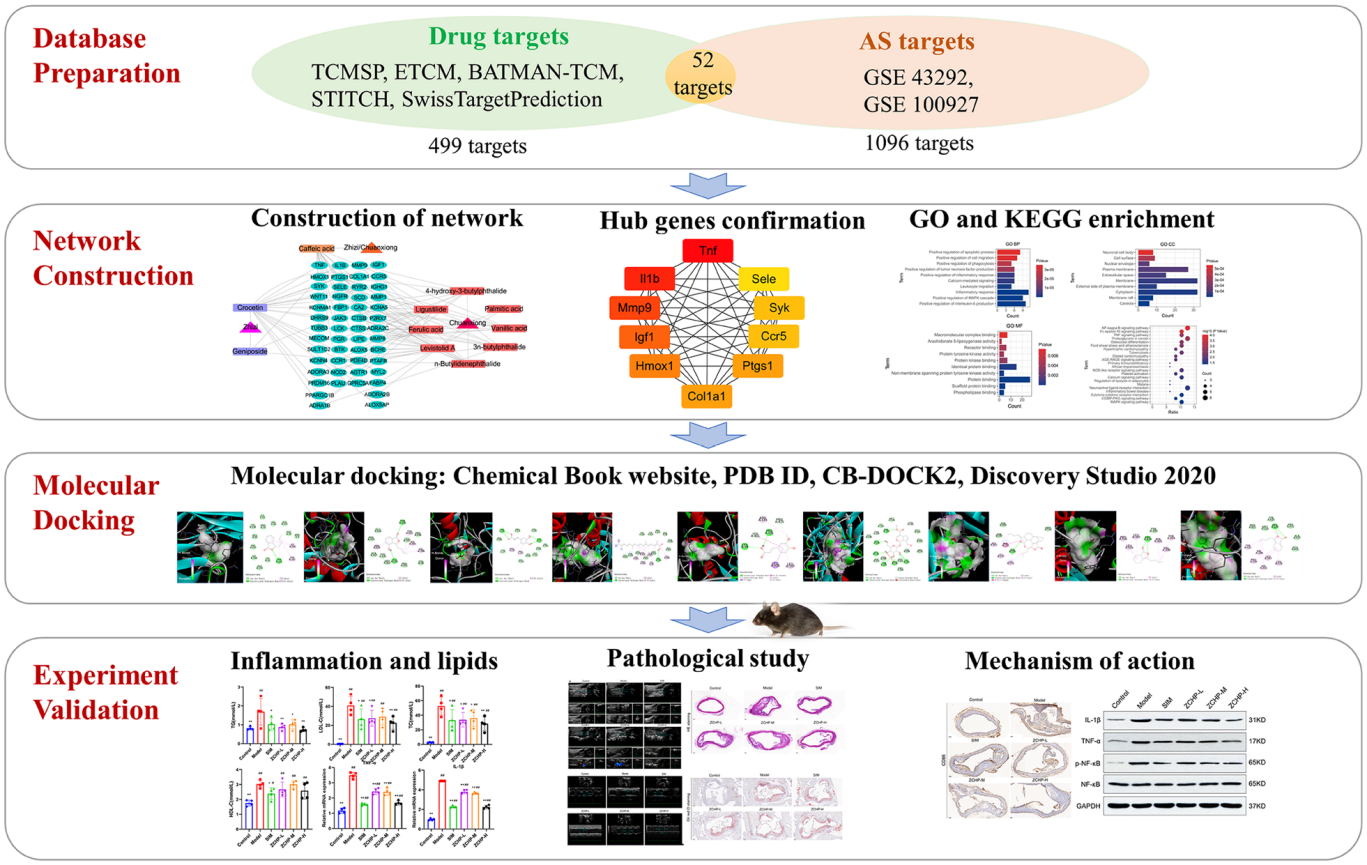

**Supplementary Information:**

The online version contains supplementary material available at 10.1186/s13020-023-00874-x.

## Background

Atherosclerosis (AS) is a kind of chronic inflammatory disease that is a general pathological basis for cardiovascular disease (CVD) [1]. Recent studies have emphasized the key role of macrophage polarization in AS progression and regression [2, 3]. M1 macrophage polarization induces the release of inflammatory cytokines mediating the acceleration of AS, including interleukin-6 (IL-6), interleukin-1α (IL-1α), tumor necrosis factor α (TNF-α), and interleukin-1β (IL-1β) [4]. In addition, M1 macrophages can secrete matrix metalloproteinases (MMPs), leading to extracellular matrix degradation, plaque instability, and rupture [5]. Therefore, hampering the phenotypic transformation of macrophages into M1 is a potential strategy for preventing and attenuating AS. TNF and nuclear factor-kappa B (NF-κB) signaling are critical reasons for inflammation [6, 7], and activation of the NF-κB p65 and TNF subunits accelerates the phenomenon of macrophage polarization toward M1 in AS plaques [3, 8].

Traditional Chinese medicine (TCM) is a promising treatment option for CVD. According to the TCM theory, the pathogenic characteristics of stasis and poison are consistent with the pathological mechanisms of inflammation, programmed cell death, angiogenesis, and plaque calcification in vulnerable CVD plaques [2, 9]. The Zhizi Chuanxiong herb pair (ZCHP) is a representative prescription for blood stasis removal and detoxification, which is achieved using *Gardenia jasminoides Ellis* (Zhizi) and *Ligusticum chuanxiong Hort.* (Chuanxiong) in a 4:5 weight ratio. ZCHP has multiple effects in ApoE^−/−^ mice, including anti-inflammation, apoptosis inhibition, and DNA methylation [10]. In addition, of the 18 blood-entering components of ZCHP [11], crocin and geniposide could reduce AS progression and plaque vulnerability by balancing macrophage polarization [12, 13]. Crocetin can inhibit the inflammatory response of endothelial cells and expression of NF-κB in the peripheral blood of patients with CVD [14, 15]. Various ZCHP components have been shown to postpone the AS progression by suppressing inflammatory response [16, 17].

Nevertheless, whether ZCHP attenuates AS progression and plaque vulnerability by regulating M1 macrophage polarization remains unclear. This study aimed to explore ZCHP’s potential action mechanism in the therapy of AS using integrated network pharmacology, molecular docking, and animal experiment strategy.

## Methods

### Screening of ZCHP components related targets

We searched the TCMSP (https://www.tcmsp-e.com/), STITCH (http://stitch.embl.de), SwissTargetPrediction (http://www.swisstargetprediction.ch), BATMAN-TCM (http://bionet.ncpsb.or g/batman-tcm/index.php), and ETCM (http://www.tcmip.cn/ETCM/index.php/Home/Index/) databases for the active ingredient targets of 18 prototype blood components of ZCHP previously observed using the UPLC-Q-TOF-MSE technology [11].

### Screening of AS-associated targets

Searching “atherosclerosis” in the GEO database (website: https://www.ncbi.nlm.nih.gov/gds/) yields two data sets, GSE43292 and GSE100927, which include 61 AS patients and 44 healthy individuals. Data standardization and screening of differentially expressed genes (DEGs) were performed by the limma software package (version 3.5.1) with database of GEO2R. DEGs were identified in samples of patients with AS compared with control samples. To identify genes with significant differences in expression fold-change (FC), the gene screening criteria were set with FC values (|log2FC|) > 1 and *P* < 0.05 to visualize in volcano plot.

### Component-target network and protein–protein interaction (PPI) network reconstruction

The genes intersecting the ZCHP candidate components and AS were mapped using jvenn (https://jvenn.toulouse.srae.fr/app/example.html) and visualized using Cytoscape 3.7.2. The obtained data on ZCHP potential targets were imported into STRING 12.0 website, and the PPI network of ZCHP was constructed. Subsequently, the hub genes in the PPI network were screened using “degree value” as the evaluation parameter in the Cytoscape plugin CytoHubba.

### Gene Ontology (GO) and Kyoto Encyclopedia of Genes and Genomes (KEGG) enrichment analysis

Intersecting genes were evaluated by using GO and KEGG enrichment analyses with the Database for Annotation, Visualization and Integrated Discovery version 6.8, with *P* < 0.05 as the cutoff. Hiplot (website: https://hiplot.com.cn/home/index.html) was employed to visualize the results.

### Molecular docking study for target prediction

The Chemical Book website (https://www.chemicalbook.com/ProductIndex.aspx) was the resource for the chemical structures of the ZCHP blood components detected by UPLC-Q-TOF-MSE. The basic principle was as follows: the biological species was considered *Homo sapiens*, conformational resolution was < 2.5 Å, conformational sequence was complete, small molecular ligand information was in the structural complex, and crystalline pH value was close to that of normal human physiology. The PDB ID of the core target of difference was selected from the database PDB (http://www.rcsb.org) [18]. The best-docking binding energy calculations were then obtained from the CB-Dock2 (website: https://cadd.labshare.cn/cb-dock2/php) [19]. Discovery Studio 2020 software was used to construct the 3D and 2D interaction visualization maps after docking completion [20].

### Animal study

The study used male ApoE^−/−^ mice with a C57BL/6 J background and C57BL/6 J wild-type mice (6 weeks old, 21–24 g) purchased from Sipeifu Biotechnology Company Limited (License No. SCXK (Beijing) 2019–0010; Beijing, China). The mice were kept in an animal barrier system with specific pathogen–free laboratory.

After a week of acclimatization to a standard feed, the ApoE^−/−^ mice were fed a high-fat diet (HFD; 78.85% basal diet, 21.00% fat, and 0.15% cholesterol) for 21 weeks; the C57BL/6 J mice were fed a standard chow diet for 21 weeks as the control group. Three ApoE^−/−^ and three C57BL/6 J mice were randomly selected and sacrificed after 9 weeks. Hematoxylin and eosin (H&E) staining and serum lipid levels were used to evaluate the degree of AS. The remaining 20 ApoE^−/−^ mice were divided evenly into 5 groups: model (normal saline 10 mL kg^−1^ *i.g)*, simvastatin (SIM, 2.6 mg day^−1^ kg^−1^ *i.g*), low dose ZCHP (ZCHP-L, 1.8 g day^−1^ kg^−1^ *i.g*), medium dose ZCHP (ZCHP-M, 3.6 g day^−1^ kg^−1^ *i.g*), and high dose ZCHP (ZCHP-H, 7.2 g day^−1^ kg^−1^ *i.g*). C57BL/6 J mice were the control group (normal saline 10 mL kg^−1^ *i.g*). At the end period of the test, the mice were fasted overnight to avoid food affecting the experimental results. Before sampling, the mice’s aortic arch and left common carotid artery (LCCA) were examined using an ultrasound device. After inhaling isoflurane anesthesia (concentration 2%, flow rate 10 mL/min), blood samples from the retroorbital venous plexus were collected and centrifuged to obtain the serum. Aortic and cardiac tissues were collected after systemic perfusion with 20 mL PBS.

### Ultrasonography

An ultrasound biomicroscopy Vevo 2100 (Visual Sonics; Toronto, Ontario, Canada) equipped with a 45-MHz, 9.0-mm depth MS550D high-frequency probe was used to measure the diastolic diameter (Dd), systolic diameter (Ds), and inner media thickness (IMT) of the aortic arch and pulse wave velocity (PWV) of the LCCA [8]. The morphology and stiffness of vascular were monitored by ultrasonography. Arterial stiffness was evaluated using PWV and the difference between the end-diastolic and end-systolic diameters (Dd-Ds). The IMT value was calculated to represent the AS burden. TP and TD time (from the tip of the QRS complex wave crest to the beginning of the Doppler waveform) were measured at the proximal and distal ends of the LCCA, and the distance (S) between the proximal and distal ends of LCCA was measured. The formula for calculating carotid PWV was PWV = S / [TD − TP (cm/s)]. M-mode ultrasonography was used to measure the IMT (distance from the aortic intima’s inner surface to the media’s outer surface).

### Histopathological, immunohistochemistry, and serum lipids analysis

The hearts were embedded in OCT tissue-freezing medium. The aortic sinus was observed under the microscope and serially sectioned (8 μm) for Oil Red O staining, which was used to identify aortic lipid-rich plaques. The aortic arches were fixed in 4% paraformaldehyde, dehydrated, embedded in paraffin wax, and sliced into 6-μm widths for H&E staining, which highlights the plaque lesion area. For immunohistochemical analysis, the sections were treated with 3% H_2_O_2_ in methanol for 30 min to cut off endogenous peroxidase activity, after that were incubated with 3% bovine serum albumin for 30 min. After washing in PBS, the sections were incubated with anti-CD86 primary antibodies (1:100, ab269587, Abcam; Cambridge, UK) to analyze macrophage expression. After a massive cleaning, the sections were incubated with biotinylated goat anti-rabbit IgG HRP (1:1000, ab6721, Abcam) for 50 min. Immunocomplexes were detected using the DAB substrate. Histopathological and immunological images were quantified using Image-Pro Plus 6.0 software (Media Cybernetics Inc., Rockville, MD, USA). The low-density lipoprotein cholesterol (LDL-C), triglyceride (TG), total cholesterol (TC), and high-density lipoprotein cholesterol (HDL-C) levels were measured using the automatic biochemical AU480 system (Beckman Coulter; Brea, CA, USA). Because the LDL-C and TC levels exceeded the linear detection range, the sera were transferred to new tubes and diluted with normal saline (1:8) to detect TG and HDL-C without dilution [21].

### Real-time quantitative reverse transcription–polymerase chain reaction

Total RNA of mice were extracted from the aortic tissue samples with Total RNA Extraction Kit (#GPQ1801, Genepool Biotechnology, Beijing, China), following the manufacturer’s instructions. Using *GAPDH* as the reference gene, reverse transcription was performed by mRNA cDNA Synthesis Kit. Relative quantitative data were calculated on a LineGene 9600Plus fluorescence quantitative polymerase chain reaction (PCR) instrument using the 2^−△△CT^ method. The summary of the primer sequences are presented in Table 1.

**Table 1:** Summary of the primer sequences for PCR analysis

Gene	Forward primer (5′–3′)	Reverse primer (5′–3′)
IL-1β	CGCAGCAGCACATCAACAAGAGC	TGTCCTCATCCTGGAAGGTCCACG
TNF-α	CCCTCCAGAAAAGACACCATG	GCCACAAGCAGGAATGAGAAG
GAPDH	AGGTCGGTGTGAACGGATTTG	TGTAGACCATGTAGTTGAGGTCA

### Western blot analysis

Protein Extraction Kit was employed to isolate the protein according to the manufacturer’s instructions. Following separation using sodium dodecyl sulfate–polyacrylamide gel electrophoresis, the samples were transferred to a 0.22-μm PVDF membrane. The membranes were incubated with the primary antibody at 4 degree celsius overnight. Primary antibodies targeting IL-1β (#26,048–1-AP, 1:500), TNF-α (#60,291–1-AP, 1:500), and NF-κB P65 (#10,745–1-AP, 1:800) were obtained from by Proteintech (Wuhan, China), whereas anti-phospho-NF-κB p65 antibody (#3033, 1:500) was derived from Cell Signaling Technology. The PVDF membranes and horseradish peroxidase–conjugated secondary antibodies were incubated for 50 min with gentle shaking. The protein bands were visualized by an ECL kit (#GPP1824; GenePool Biotechnology, Beijing, China). Finally, densitometry values of the bands were obtained using Quantity One version 4.6.2.

### Statistical analysis

The software SPSS was utlized to do the statistical analysis. Means ± standard deviation (SD; X ± S) are presented for measurement data. Mutual comparisons of multiple groups were performed with single factor analysis of variance. For the post-hoc test, the LSD test was used to test data with homogeneity of variance, Games-Howell test was used to examine data with heterogeneity of variance, respectively. It was considered statistically significant when *P* < 0.05.

## Results

### Screening of ZCHP components-related targets

To identify ZCHP’s active components and their potential anti-AS targets, we used ZCHP blood components according to the screening criteria of ADME parameters (GI = high, DL index ≥ 3 Yes) [22, 23]. In addition, despite SwissADME not being reported or having poor GI absorption, crocetin [14, 24], geniposide [12, 25], and levistilide A [26, 27] reportedly have high pharmacological activity and were brought into the network analysis in the next step. Finally, 13 bioactive components were identified as the candidate ingredients (Additional file 1: Table S1).

### Screening of AS-related targets

**Fig. 1:**
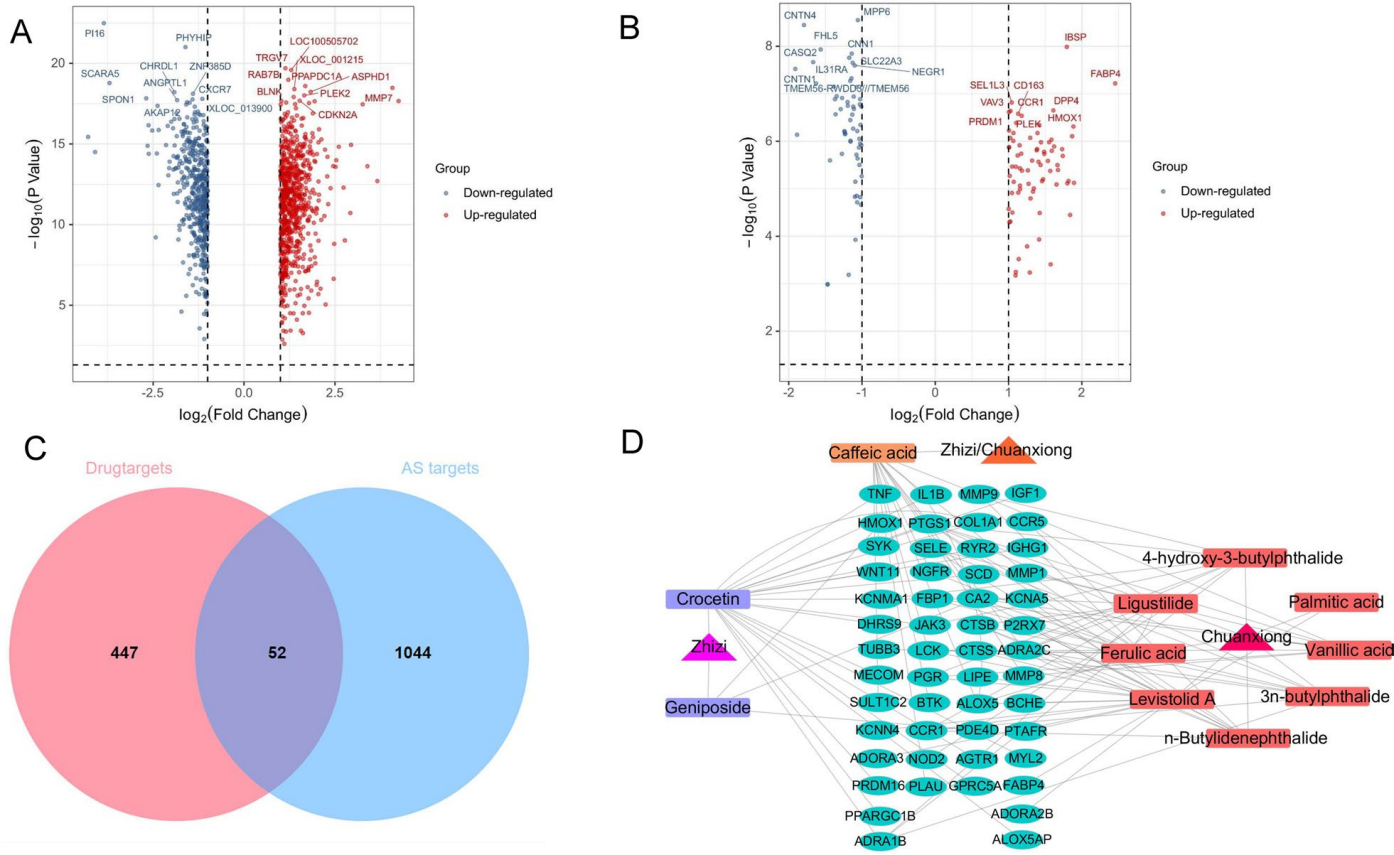
Identification of ZCHP candidate targets for AS treatment. **A** Volcano plot representing the significance and fold-change of differentially expressed genes extracted from GSE46394 and **B** GSE100927. Both red probes represent highly expressed genes (log2FC > 1, *P* < 0.05); blue probes represent lower expression genes (log2FC <  − 1, *P* < 0.05); **C** Venn diagram of ZCHP and AS targets; **D** Construction of the ZCHP-components-targets-AS network. Triangular nodes representing the two herbs in ZCHP, rectangular nodes representing components, and elliptical nodes representing targets

We obtained 117 and 1,034 differentially expressed genes from the GSE43292 and GSE100927 databases, respectively; 1,096 genes remained after duplicate removal. In the volcano plot (Fig. 1A, B), genes in red are highly expressed, whereas genes in blue are expressed at low levels.

### Network of ZCHP components-targets-AS

Except the senkyunolide I and H components, which had no intersection with AS targets, the remaining 11 components and 52 targets were used to construct the ZCHP components-targets-AS network (Fig. 1C). The network had 66 nodes and 116 edges, including triangle nodes representing two herbs in ZCHP, rectangle nodes representing the 11 blood components, and elliptical nodes representing targets. (Fig. 1D and Additional file 1: Table S2).

### The PPI network and functional enrichment of candidate targets

**Fig. 2:**
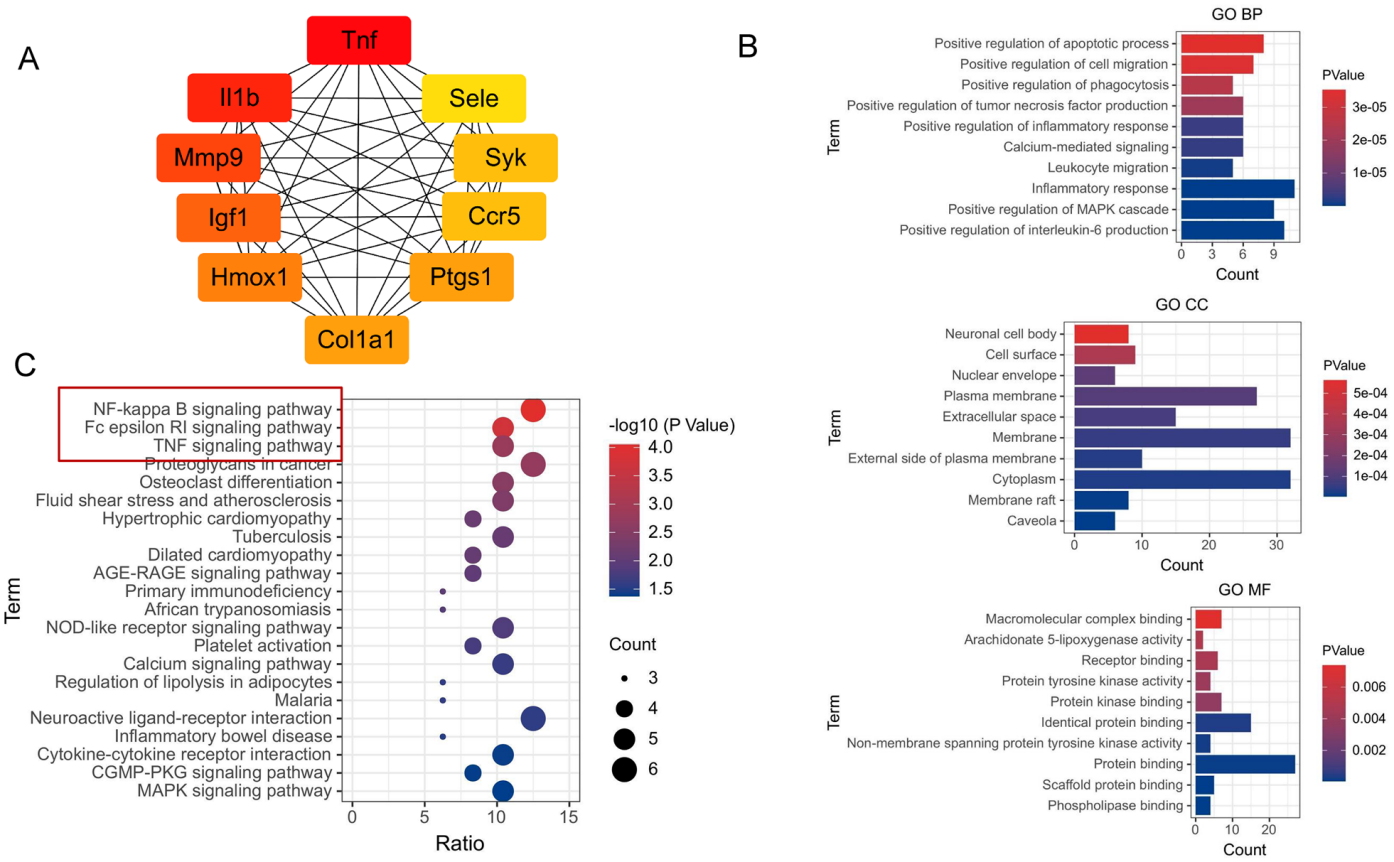
PPI network and functional enrichment analyses of ZCHP action targets in AS treatment. **A** The PPI network of the ZCHP action targets. The nodes indicate targets, the redder the color, the stronger the interaction. **B** The top 30 enriched GO biological process (BP), GO cell component (CC), and GO molecular function (MF) terms of ZCHP candidate targets. **C** The top 22 enriched KEGG pathways of ZCHP candidate targets. The x-coordinate is for the ratio, the circle size indicates the number of genes enriched, the y-coordinate is for the KEGG pathways, and the different colors represent adjusted *P* values

Using the CytoHubba Degree plugin, 10 hub proteins in the network, which were TNF, IL-1β, IGF1, MMP9, COL1A1, CCR5, HMOX1, PTGS1, SELE, and SYK, had scores ≥ 27. TNF (scores = 45) and IL-1β (scores = 43) were highlighted as the two main targets of ZCHP for AS anti-inflammatory effects (Additional file 1: Table S3). As shown in Fig. 2A, 52 core targets were input to the DAVID 6.8 database for GO and KEGG enrichment analyses (*P* < 0.05). The analysis identified 230 GO terms and 22 KEGG signaling pathways, including inflammatory response, collagen catabolic process, positive regulation of interleukin-6 and NF-κB, Fc epsilon RI, and TNF signaling pathways. Among them, the NF-κB signaling pathway showed the strongest relationship (*P* = 8.91 × 10^–5^, gene count = 6; Fig. 2B, C).

### Molecular docking

**Fig. 3:**
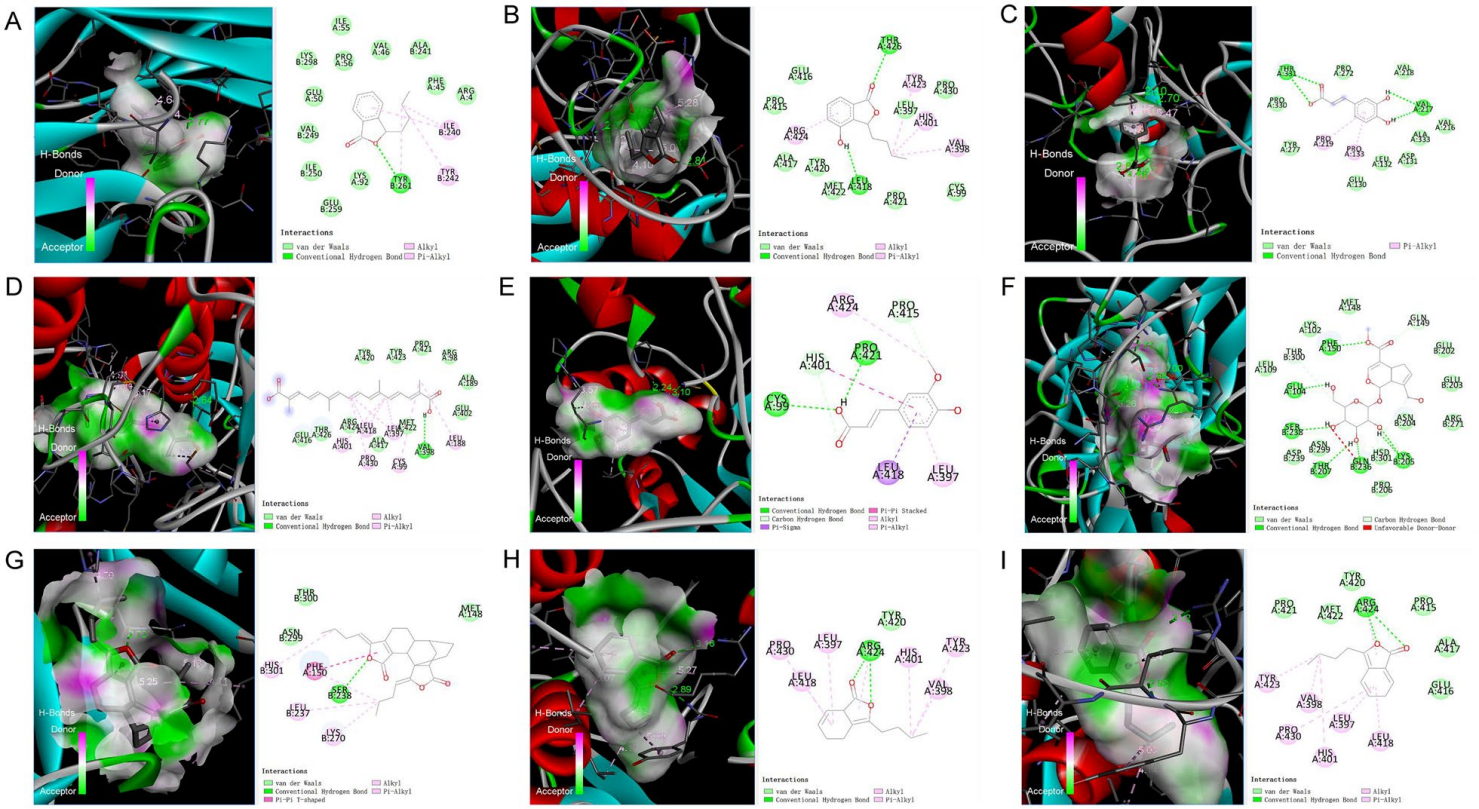
The molecular docking models of targets and components. **A** 3-n-butylphthalide and IL-1β; **B** 4-Hydroxy-3-butylphthalide and MMP9; **C** caffeic acid and MMP9; **D** crocetin and MMP9; **E** ferulic acid and MMP9; **F** geniposide and IL-1β; **G** levistilide A and IL-1β; **H** ligustilide and MMP9; **I** N-butylidenephthalide and MMP9. The dotted green lines represent hydrogen bonds, the pink color represents an alkyl/π-alkyl interaction, and the light green circle represents the van der Waals force

Based on the PPI hub network results, CB-Dock2 database was used for molecular docking analysis (TNF-PDB ID: 2AZ5, MMP9-PDB ID: IL6J, IL-1β-PDB ID: 4GAF, IGF1-PDB ID: 2DSQ, CCR5-PDB ID: 5YD3, HMOX1-PDB ID: 1NI6, PTGS1-PDB ID: 6Y3C, SELE-PDB ID: 1G1T, COL1A1-PDB ID: 5CTI, SYK-PDB ID: 4FL1); the binding energy are shown in Additional file 1: Table S4. According to the docking principle, if the energy is <  − 7.0 kcal/mol, the components have a strong affinity with the targets. We observed that only 9 ZCHP components have strong binding energies with 10 core targets; their 3D and 2D binding structures are shown in Fig. 3. Overall, the ligands and receptors were bound mainly by hydrogen bonds. The secondary effects were van der Waals and alkyl/π-alkyl interactions. Among the molecules forming the best binding energies were 3-n-butylphthalide with IL-1β (− 7.8 kcal/mol), 4-hydroxy-3-butylphthalide with MMP9 (− 7.7 kcal/mol), caffeic acid with MMP9 (− 7.7 kcal/mol), crocetin with MMP9 (− 8.0 kcal/mol), ferulic acid with MMP9 (− 7.3 kcal/mol), geniposide with IL-1β (− 8.4 kcal/mol), levistilide A with IL-1β (− 9.7 kcal/mol), ligustilide with MMP9 (− 7.6 kcal/mol), and N-butylidenephthalide with MMP9 (− 7.9 kcal/mol). The docking results show that the most potent levistilide A target was IL-1β, which provides a reference for follow-up experimental verification.

### Animal model validation

#### Confirmation of AS formation after a high-fat diet for 9 weeks in ApoE^−/−^ mice

**Fig. 4:**
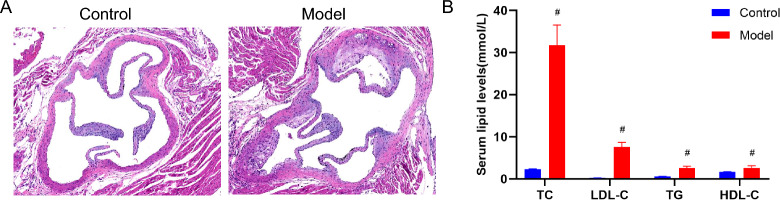
Confirmation of AS formation after a HFD in ApoE^−/−^ mice. **A** Representative image of H&E for evaluating plaque formation (n = 3 per group, scale bars = 100 µm); **B** Serum levels of TC, LDL-C, TG, and HDL-C (n = 3 per group) ^#^ *P* < 0.05 vs. control

H&E staining was applied to the aortic roots of ApoE^−/−^ and C57BL/6 J mice to identify AS. Contrasted with the aortas of C57BL/6 J mice, those of ApoE^−/−^ mice showed significant plaque formation (Fig. 4A). Furthermore, LDL-C, TC, and TG levels of model group were significantly greater than those of control group after 9 weeks of HFD feeding (Fig. 4B). Therefore, AS developed in ApoE^−/−^ mice after HFD feeding for 9 weeks.

#### Effect of ZCHP on arterial stiffness and atherosclerotic load in ApoE−/− mice

**Fig. 5:**
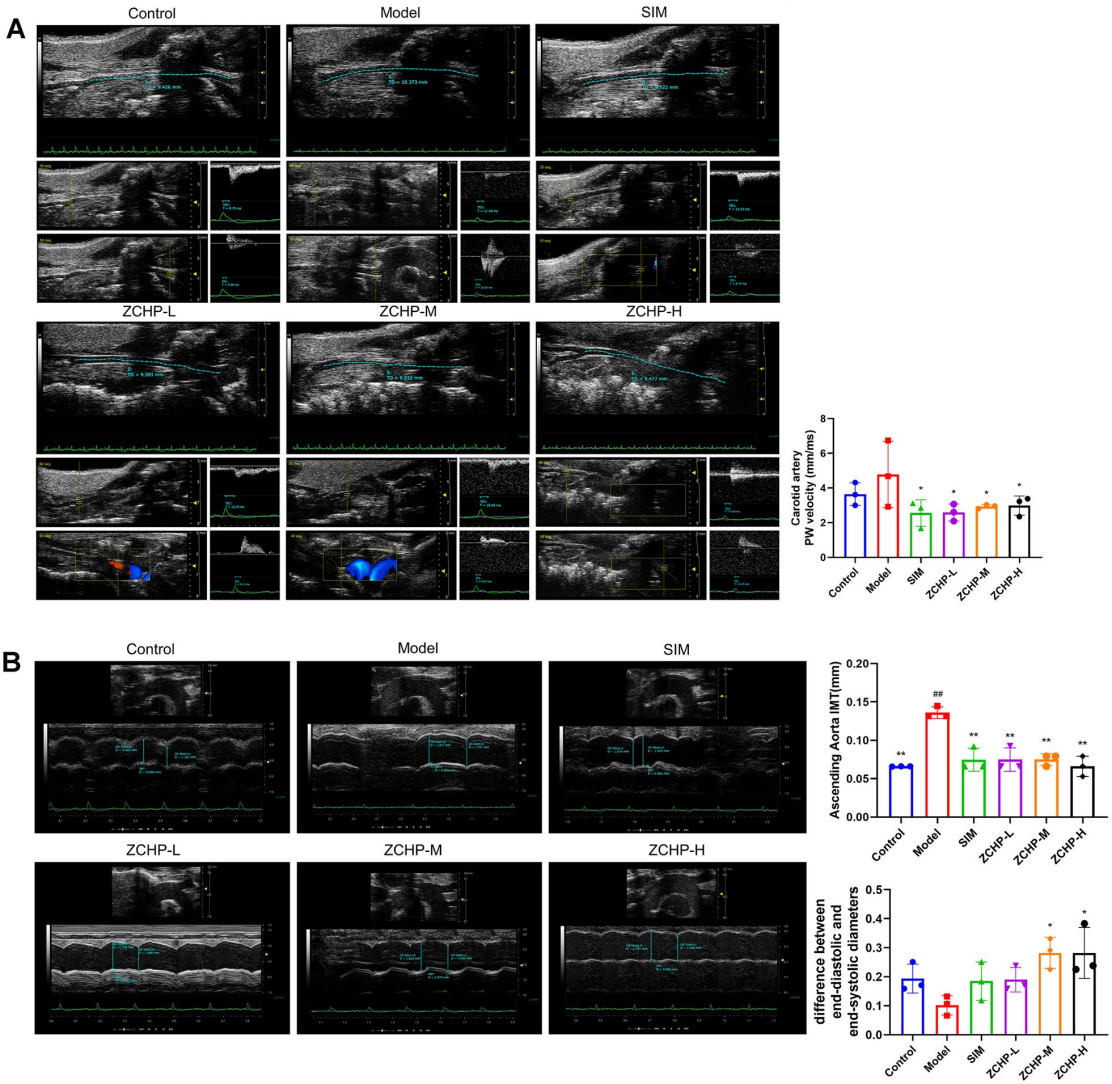
ZCHP reduces arterial stiffness and atherosclerotic load in ApoE^−/−^ mice. **A** Representative image of PWV of the LCCA and quantitative analysis (n = 3 per group). **B** Representative image of IMT and the difference between end-diastolic and end-systolic diameters of the aortic arch and quantitative analysis (n = 3 per group). * *P* < 0.05 and ** *P* < 0.01 vs. model; ^#^ *P* < 0.05 and ^##^ *P* < 0.01 vs. control

As shown in Fig. 5A, B, PWV and IMT were obviously greater in the model group than that of control group. These effects were markedly reversed in the three ZCHP dose groups. Furthermore, Dd-Ds levels were noticeably lower in the model group than that in the control group. The ZCHP-M and -H groups showed a significantly increased of Dd-Ds levels after compared with the model group. These results suggested that ZCHP can reduce the hardness of the LCCA and IMT of the aortic arch in ApoE^−/−^ mice.

#### ZCHP attenuates atherosclerosis in ApoE−/− mice

**Fig. 6:**
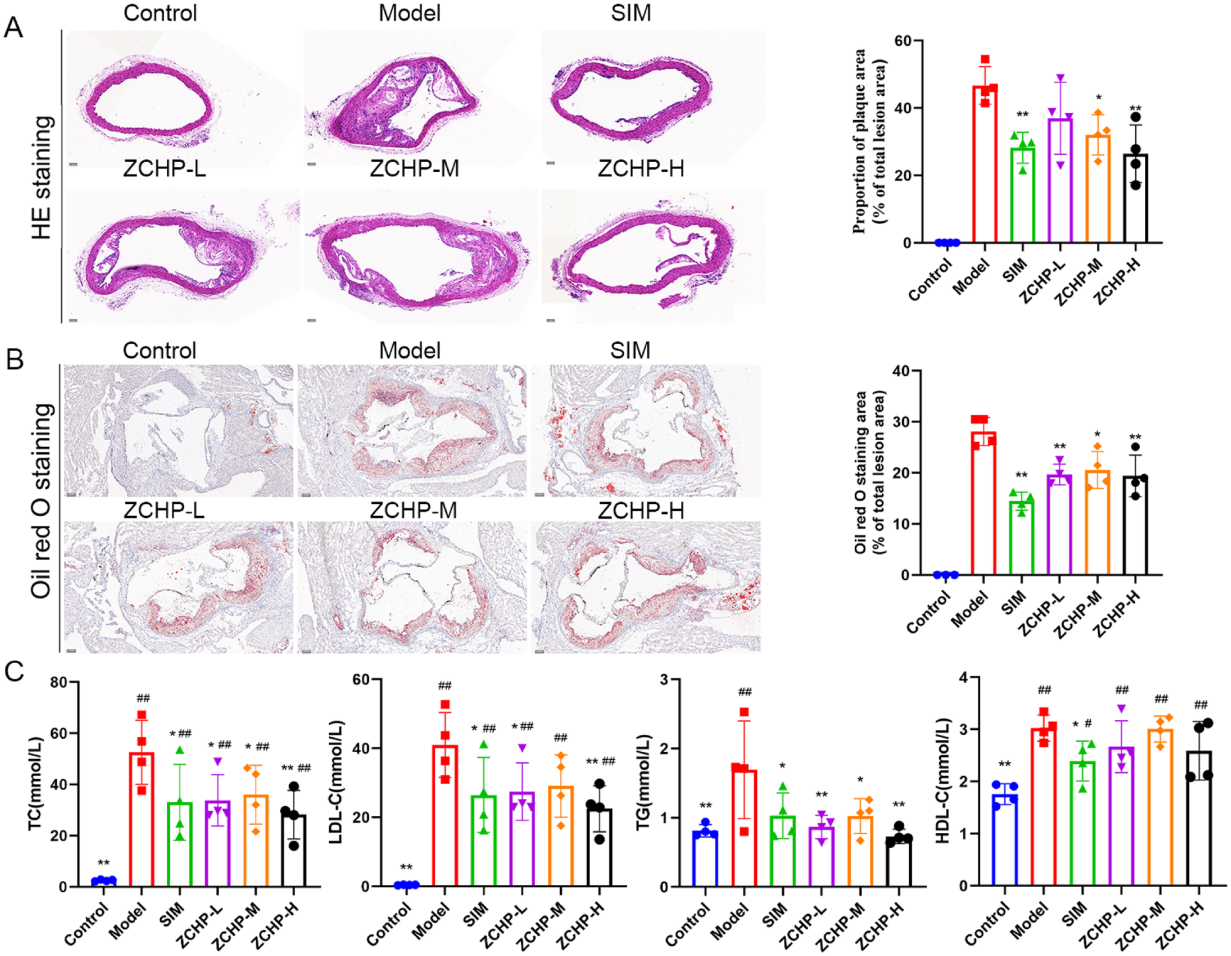
ZCHP reduces atherosclerotic lesions in ApoE^−/−^ mice. **A** Typical HE-stained image of the aortic arch and quantitative analysis (n = 4 per group, scale bars: 225 µm); **B** Typical aortic sinus image of Oil Red O staining and quantitative analysis (n = 4 per group, scale bars: 100 µm); **C** The effects of ZCHP on TC, LDL-C, TG, and HDL-C levels in serum samples (n = 4 per group). * *P* < 0.05 and ** *P* < 0.01 vs. model; ^#^ *P* < 0.05 and ^##^ *P* < 0.01 vs. control

H&E staining revealed that the plaque area was remarkably increased in the model group (*P value* < 0.05). Contrasted with the model group, ZCHP-M and -H groups had significantly reduced the aortic arch plaque area (Fig. 6A). Oil Red O staining of the aortic sinus showed no red lipid plaques in the aortic sinus of the control group. The large red lipid plaque was observed on the aortic sinus in the model group. Compared with the AS model group, the ZCHP*-*treated ApoE^−/−^ mice had significantly reduced red lipid plaque in the aortic sinus. These data showed that the ZCHP-L, -M, and -H groups had ameliorated HFD-induced AS, with a more obvious effect in the ZCHP-H group (Fig. 6B).

HFD significantly increased LDL-C, TC, TG, and HDL-C levels in the model group contrasted with them in the control group. The ZCHP-H group exhibited decreased LDL-C, TC, TG, and HDL-C levels after 12 weeks of treatment. TG and TC levels markedly decreased in three ZCHP doses and SIM groups (Fig. 6C).

#### ZCHP suppresses the TNF/NF-κB axis and M1 macrophage polarization

**Fig. 7:**
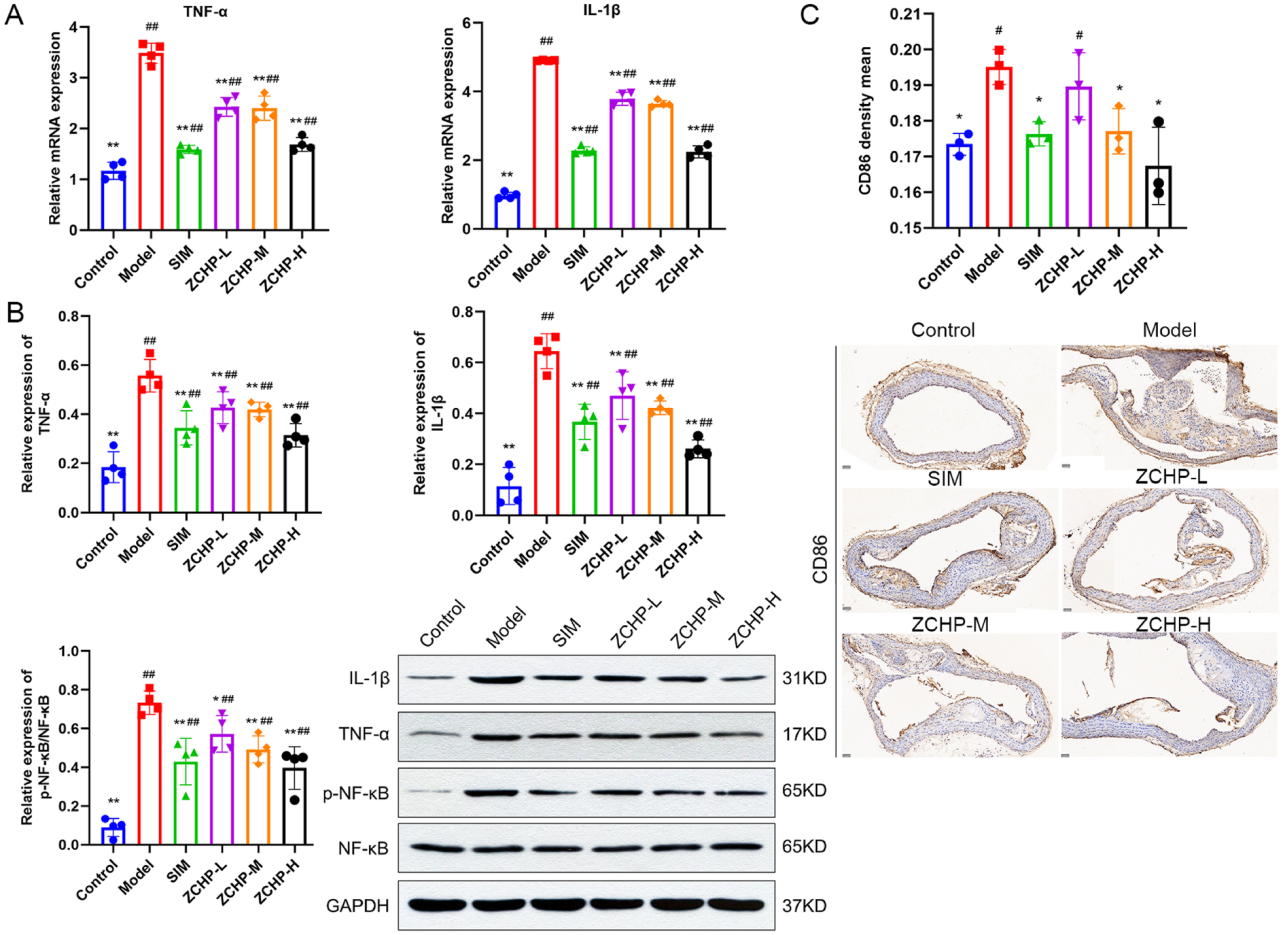
ZCHP restrained the activation of pro-inflammatory cytokines, NF-κB, and M1 macrophage polarization biomarkers. **A** The mRNA levels of TNF-α and IL-1β (n = 4 per group); **B** Representative Western blot images and relative protein levels of p-NF-κB, NF-κB, TNF-α, and IL-1β in ApoE^−/−^ mice (n = 4 per group); **C** Representative image of immunohistochemical staining shows the expression of CD86 in the aortic arch and quantitative analysis (n = 3 per group, scale bars: 400 µm). * *P* < 0.05 and ** *P* < 0.01 vs. model; ^#^ *P* < 0.05 and ^##^ *P* < 0.01 vs. control

The network pharmacological analysis showed that the core targets of ZCHP were enriched mainly in TNF and NF-κB signaling pathways. Therefore, we focused on exploring the expression levels of TNF-α, IL-1β, and NF-κB-related targets in the TNF signaling pathway. The mRNAs of IL-1β and TNF-α were obviously upregulated in the model group, and ZCHP reversed this phenomenon (Fig. 7A). Protein levels were verified to be consistent; ZCHP reduced TNF-α and IL-1β protein expression.

The expression of NF-κB signaling pathway in aortic tissues was detected to evaluate its relationship with AS (Fig. 7B). Notably, the three ZHCP doses groups showed that ZCHP can significantly inhibit p-NF-κB and NF-κB protein expression levels. Because IL-1β, TNF-α, and NF-κB pathways are closely related to M1 macrophage polarization, CD86 antibody levels, a marker of M1 macrophage polarization, were examined using immunohistochemical staining of the aortic arch. Significantly greater CD86 levels were observed in the model group than in the control group. The expression of the M1 macrophage polarization marker was inhibited in the ZCHP-M and -H groups, which was not in ZCHP-L group (Fig. 7C).

## Discussion

Atherosclerotic diseases may be caused by lipid accumulation in the blood vessels and inflammatory immune responses [28]. Since ancient times, TCM has been used to prevent and treat disease based on multiple systematic, targets, and components approach [29]. This study combined network screening, computer simulation docking, and in vivo experiment to explore the targets and potential mechanisms of ZCHP anti-AS activity and found that ZCHP may significantly affect AS.

The three ZCHP doses groups showed decreased levels of IMT in the aortic arch and PWV in the LCCA, and Dd-Ds were increased in ZCHP-M and ZCHP-H, indicating that ZCHP can improve the degree of AS. PWV and the difference between the end-diastolic and end-systolic diameters relate to vascular stiffness: the stiffer the blood vessel, the faster the conduction velocity [30]. PWV is a strong predictor of CVDs in atherosclerotic populations [31]. Meanwhile, IMT can relate better to the degree of AS lesions [32]. Furthermore, histological analysis showed that ZCHP reduced the aortic plaque area and Oil Red O staining area in ApoE^−/−^ mice. Lipid deposition increases the risk factors for AS progression; nevertheless, ZCHP decreased the serum levels of LDL, TC, and TG in HFD-feeding ApoE^−/−^ mice. Pharmacodynamic experiments showed that ZCHP not only lowered serum lipid levels, but also improved vascular stiffness and reduced plaque area. These observations indicate that ZCHP is a potential strategy for treating AS. However, the mechanisms underlying ZCHP’s effects in AS remain largely unknown.

In this study, 11 candidate components of ZCHP were identified using network pharmacological analysis, and 52 potential targets of ZCHP for AS treatment were evaluated. Network pharmacology establishes a “complex-target/targeted disease” network to predict the mechanism of small-molecule regulation of disease using high data throughput. It has become a pharmacological tool for studying the multiple components and targets in the complex TCM system [33]. PPI network analysis further showed that the core targets of ZCHP against AS include TNF, IL-1β, IGF1, MMP9, COL1A1, CCR5, HMOX1, PTGS1, SELE, and SYK. Among them, TNF and IL-1β are the core targets of NF-κB and TNF signaling pathways. According to KEGG analysis, 22 signaling pathways were highly enriched, including the NF-κB, Fc epsilon RI, and TNF signaling pathways. Molecular docking analysis was performed to explore ZCHP’s mechanism of action in AS. Molecular docking studies showed high binding energy between ZCHP components and the inflammation-related factors IL-1β and MMP9. These results suggest that ZCHP blood components strongly interact with inflammatory signaling pathways TNF and NF-κB and core target IL-1β, which are considered hubs of inflammation regulation and macrophage polarization.

Activated macrophages are critical for the progression of AS and vulnerable to plaque [34]. Currently, regulation of the M1–M2 macrophage polarization balance is an emerging therapeutic target for various inflammatory diseases [35]. Under the patch microenvironment’s influence, macrophages polarization was taken place and transform into pro-inflammatory M1-type macrophages, promoting late plaque rupture [3]. M1 macrophages release mainly IL-6, TNF, IL-1β, and else inflammatory factors. Among them, TNF-induced NF-κB activation is a key point in the M1 macrophage response [6]**.** NF-κB regulates the polarization of macrophages and induces the production of these inflammatory factors, suggesting that the NF-κB signaling pathway is indispensable for activating M1 macrophages [6, 8, 36–38]. Therefore, the expression of the polarization marker CD86 was examined in M1 macrophages. After ZCHP intervention, the IL-1β, TNF-α, and CD86 levels were significantly declined in ApoE^−/−^ mice (Fig. 7), suggesting that ZCHP inhibits the transformation of plaque macrophages into the M1-type. A Western blot study shows that ZCHP noticeably restrained the activation of NF-κB of ApoE^−/−^ mice, suggesting that ZCHP inhibits the progression of AS by regulating the TNF/NF-κB axis. These data indicate that ZCHP can inhibit TNF/NF-κB axis-mediated M1 macrophage polarization.

## Conclusion

Candidate targets of ZCHP for AS treatment was identified via combining network screening, computer simulation docking and in vivo experiment. This study demonstrated that ZCHP significantly inhibited AS progression; the underlying mechanism may be inhibiting M1 macrophage polarization in AS plaques, partially via the TNF/NF-κB axis.

### Supplementary Information

**Additional file 1: Table S1.** ZCHP blood components according to ADME parameters. **Table S2.** Construction of network of ZCHP-components-targets-AS. **Table S3.** Top 10 targets scores in network PPI ranked by degree method. **Table S4.** Molecular docking scores.

## Data Availability

The original contributions of current study are included in this article and additional files.

## Abbreviations

AS: Atherosclerosis
CVD: Cardiovascular disease
Dd: Diastolic diameter of the aortic arch
Dd-Ds: Difference between the end-diastolic and end-systolic diameters
Ds: Systolic diameter of the aortic arch
FC: Fold-change
HDL-C: High-density lipoprotein cholesterol
HFD: High-fat diet
H&E: Hematoxylin and eosin
IL: Interleukin
IMT: Inner media thickness
KEGG: Kyoto Encyclopedia of Genes and Genomes
LCCA: Left common carotid artery
LDL-C: Low-density lipoprotein cholesterol
MMPs: Matrix metalloproteinases
PPI: Protein–protein interaction
PWV: Pulse wave velocity
TC: Total cholesterol
TCM: Traditional Chinese medicine
TNF: Tumor necrosis factor
ZCHP: Zhizi Chuanxiong herb pair
