# Supplementary material for: Effects and mechanisms of Zhizi Chuanxiong herb pair against atherosclerosis: an integration of network pharmacology, molecular docking, and experimental validation
Source: Chin Med. 2024 Jan 11;19:8. doi: 10.1186/s13020-023-00874-x (PMC10782628; doi:10.1186/s13020-023-00874-x)
Supplement: Supplementary file 1 — Additional file 1: Table S1. ZCHP blood components according to ADME parameters. Table S2. Construction of network of ZCHP-components-targets-AS. Table S3. Top 10 targets scores in network PPI ranked by degree method. Table S4. Molecular docking scores. [file 13020_2023_874_MOESM1_ESM.docx]

**Additional Table 1** ZCHP blood components according to ADME parameters

| No. | Components | Molecular Formulas | Category | GI absorption | Druglikeness | Bioavailability Score |
| --- | --- | --- | --- | --- | --- | --- |
| 1 | 3n-butylphthalide | C_12_H_14_O_2_ | Phthalide | High | 4 Yes* | 0.55 |
| 2 | 4-hydroxy-3-butylphthalide | C_12_H_14_O_3_ | Phthalide | High | 5 Yes* | 0.55 |
| 3 | Caffeic acid | C_9_H_8_O_4_ | Organic acid | High | 4 Yes* | 0.56 |
| 4 | Crocetin | C_20_H_24_O_4_ | Diterpenoid | Unknwon | Unknwon | Unknwon |
| 5 | Ferulic acid | C_10_H_10_O_4_ | Organic acid | High | 4 Yes* | 0.85 |
| 6 | Geniposide | C_17_H_24_O_10_ | Iridoid | Low | 1 Yes* | 0.11 |
| 7 | Levistolid A | C_24_H_28_O_4_ | Phthalide | Unknwon | Unknwon | Unknwon |
| 8 | Ligustilide | C_12_H_14_O_2_ | Lactone | High | 4 Yes* | 0.55 |
| 9 | n-Butylidenephthalide | C_12_H_12_O_2_ | Phthalide | High | 4 Yes* | 0.55 |
| 10 | Palmitic acid | C_16_H_32_O_2_ | Organic acid | High | 3 Yes | 0.85 |
| 11 | Vanillic acid | C_8_H_8_O_4_ | Organic acid | High | 4 Yes* | 0.85 |
| 12 | Senkyunolide H | C_12_H_16_O_4_ | Lactone | High | 5 Yes* | 0.55 |
| 13 | Senkyunolide I | C_12_H_16_O_4_ | Lactone | High | 5 Yes* | 0.55 |

**Notes:** Gastrointestinal absorption (GI) in Pharmacokinetics obtained from SwissADME. Druglikeness (DL) also obtained

from SwissADME, including Lipinski, Ghose, Veber, Egan, and Muegge rules. ^*^The numbers in the DL mean how many times

“Yes; 0 violations” has appeared in Lipinski 5 rules.

**Additional Table 2** Construction of network of ZCHP-components-targets-AS



| No. | Components | Targets | No. | Components | Targets |
| --- | --- | --- | --- | --- | --- |
| 1 | Palmitic acid | FABP4 | 54 | Ferulic acid | CA2 |
| 2 | 3n-butylphthalide | PTGS1 | 55 | Ferulic acid | ALOX5 |
| 3 | 3n-butylphthalide | ADRA2C | 56 | Ferulic acid | MMP9 |
| 4 | 3n-butylphthalide | ADRA1B | 57 | Ferulic acid | MMP1 |
| 5 | 3n-butylphthalide | SELE | 58 | Ferulic acid | PTGS1 |
| 6 | 3n-butylphthalide | CTSS | 59 | Ferulic acid | LCK |
| 7 | 3n-butylphthalide | CTSB | 60 | Ferulic acid | NGFR |
| 8 | 3n-butylphthalide | P2RX7 | 61 | Ferulic acid | TUBB3 |
| 9 | 3n-butylphthalide | CCR1 | 62 | Ferulic acid | FBP1 |
| 10 | 4-hydroxy-3-butylphthalide | PTGS1 | 63 | Geniposide | CA2 |
| 11 | 4-hydroxy-3-butylphthalide | ADRA2C | 64 | Geniposide | HMOX1 |
| 12 | 4-hydroxy-3-butylphthalide | ADRA1B | 65 | Geniposide | PDE4D |
| 13 | 4-hydroxy-3-butylphthalide | MMP9 | 66 | Levistolid A | PGR |
| 14 | 4-hydroxy-3-butylphthalide | KCNMA1 | 67 | Levistolid A | CTSB |
| 15 | 4-hydroxy-3-butylphthalide | PGR | 68 | Levistolid A | CCR1 |
| 16 | 4-hydroxy-3-butylphthalide | JAK3 | 69 | Levistolid A | ADORA3 |
| 17 | 4-hydroxy-3-butylphthalide | NOD2 | 70 | Levistolid A | P2RX7 |
| 18 | Caffeic acid | BTK | 71 | Levistolid A | PTAFR |
| 19 | Caffeic acid | ADRA2C | 72 | Levistolid A | ALOX5 |
| 20 | Caffeic acid | PLAU | 73 | Levistolid A | BCHE |
| 21 | Caffeic acid | TNF | 74 | Levistolid A | CTSS |
| 22 | Caffeic acid | ALOX5 | 75 | Levistolid A | ADORA2B |
| 23 | Caffeic acid | SULT1C2 | 76 | Levistolid A | LCK |
| 24 | Caffeic acid | CA2 | 77 | Levistolid A | KCNA5 |
| 25 | Caffeic acid | ALOX5 | 78 | Levistolid A | LIPE |
| 26 | Caffeic acid | MMP9 | 79 | Levistolid A | KCNN4 |
| 27 | Caffeic acid | MMP1 | 80 | Ligustilide | ADRA2C |
| 28 | Caffeic acid | NGFR | 81 | Ligustilide | ADRA1B |
| 29 | Caffeic acid | SYK | 82 | Ligustilide | CA2 |
| 30 | Caffeic acid | LCK | 83 | Ligustilide | PTGS1 |
| 31 | Caffeic acid | PTGS1 | 84 | Ligustilide | CTSB |
| 32 | Caffeic acid | MMP8 | 85 | Ligustilide | P2RX7 |
| 33 | Crocetin | ADRA1B | 86 | Ligustilide | ADRA2C |
| 34 | Crocetin | IGHG1 | 87 | Ligustilide | SELE |
| 35 | Crocetin | COL1A1 | 88 | Ligustilide | CCR1 |
| 36 | Crocetin | IGF1 | 89 | Ligustilide | CCR5 |
| 37 | Crocetin | DHRS9 | 90 | n-Butylidenephthalide | CA2 |
| 38 | Crocetin | GPRC5A | 91 | n-Butylidenephthalide | PTGS1 |
| 39 | Crocetin | JAK3 | 92 | n-Butylidenephthalide | CCR1 |
| 40 | Crocetin | WNT11 | 93 | n-Butylidenephthalide | CCR5 |
| 41 | Crocetin | MECOM | 94 | n-Butylidenephthalide | P2RX7 |
| 42 | Crocetin | PTGS1 | 95 | n-Butylidenephthalide | ADRA2C |
| 43 | Crocetin | IL1B | 96 | n-Butylidenephthalide | CTSB |
| 44 | Crocetin | ALOX5AP | 97 | n-Butylidenephthalide | SELE |
| 45 | Crocetin | SCD | 98 | Vanillic acid | PTGS1 |
| 46 | Crocetin | MYL2 | 99 | Vanillic acid | CA2 |
| 47 | Crocetin | PRDM16 | 100 | Vanillic acid | LCK |
| 48 | Crocetin | AGTR1 | 101 | Vanillic acid | FBP1 |
| 49 | Crocetin | TNF | 102 | Vanillic acid | MMP9 |
| 50 | Crocetin | KCNA5 | 103 | Vanillic acid | MMP1 |
| 51 | Crocetin | RYR2 | 104 | Vanillic acid | MMP8 |
| 52 | Crocetin | PPARGC1B | 105 | Vanillic acid | PGR |
| 53 | Crocetin | COL1A1 |  |  |  |

**Additional Table 3** Top 10 targets scores in network PPI ranked by degree method

| Rank | Name | Score | Rank | Name | Score |
| --- | --- | --- | --- | --- | --- |
| 1 | TNF | 45 | 6 | COL1A1 | 29 |
| 2 | IL-1B | 43 | 7 | PTGS1 | 29 |
| 3 | MMP9 | 41 | 8 | CCR5 | 28 |
| 4 | IGF1 | 36 | 8 | SYK | 28 |
| 5 | HMOX1 | 35 | 10 | SELE | 27 |

**Additional Table 4** Molecular docking scores

| Target | PDB ID | 3-n-butylphthalide | 4-Hydroxy-3-butylphthalide | Caffeic acid | Crocetin | Crocin III | Ferulic acid | Gardenoside | Genipin 1-gentiobioside | Geniposide | Geniposidic acid | Levistilide A | Ligustilide | N-butylidenephthalide | Palmitic acid | Quinic acid | Vanillic acid |
| --- | --- | --- | --- | --- | --- | --- | --- | --- | --- | --- | --- | --- | --- | --- | --- | --- | --- |
| SYK | 4FL1 | -5.6 | -5.5 | -5.3 | -6.9 | -8.2 | -5.4 | -6.3 | -7.2 | -6.7 | -6.0 | -8.1 | -5.7 | -5.7 | -4.8 | -4.5 | -5.0 |
| SELE | 1G1T | -5.2 | -5.3 | -5.4 | -6.2 | -7.2 | -5.3 | -5.8 | -7.1 | -6.2 | -6.2 | -6.8 | -4.8 | -5.0 | -4.0 | -4.9 | -5.1 |
| MMP9 | IL6J | -7.5 | -7.7 | -7.7 | -8.0 | -7.8 | -7.3 | -7.3 | -7.5 | -6.9 | -7.2 | -8.5 | -7.6 | -7.9 | -5.9 | -7.2 | -6.8 |
| HMOX1 | 1NI6 | -7.3 | -7.3 | -6.7 | -7.5 | -8.2 | -6.6 | -8.3 | -8.8 | -7.9 | -8.2 | -8.9 | -7.2 | -7.3 | -6.4 | -6.3 | -6.0 |
| TNF | 2AZ5 | -6.6 | -6.5 | -6.2 | -7.7 | -9.1 | -6.6 | -7.0 | -8.1 | -7.7 | -7.5 | -8.7 | -6.4 | -6.5 | -5.4 | -5.6 | -5.6 |
| IGF1 | 2DSQ | -5.7 | -5.7 | -6.1 | -6.6 | -8.3 | -6.2 | -7.8 | -8.7 | -7.7 | -8.3 | -8.2 | -5.8 | -6.0 | -4.9 | -5.9 | -5.7 |
| IL1B | 4GAF | -7.8 | -7.1 | -7.0 | -7.6 | -8.3 | -6.8 | -9.3 | -10.6 | -8.4 | -10.0 | -9.7 | -7.2 | -7.3 | -3.9 | -6.8 | -6.5 |
| PTGS1 | 6Y3C | -7.2 | -7.4 | -6.7 | -7.8 | -8.8 | -6.6 | -7.2 | -8.8 | -7.2 | -7.6 | -8.3 | -7.4 | -7.5 | -5.6 | -6.6 | -6.4 |
| CCR5 | 5YD3 | -6.4 | -6.7 | -6.4 | -6.5 | -9.1 | -6.4 | -9.2 | -10.1 | -7.7 | -9.6 | -7.6 | -6.1 | -6.4 | -6.1 | -4.2 | -6.7 |
| COL1A1 | 5CTI | -6.1 | -5.9 | -5.8 | -6.7 | -7.6 | -5.9 | -6.4 | -6.1 | -5.9 | -5.9 | -7.6 | -6.1 | -6.1 | -5.3 | -5.3 | -4.7 |
